# Tobacco-related toxicant exposure among people with and without experience of psychosis: findings from the US Population Assessment of Tobacco and Health study

**DOI:** 10.1136/bmjopen-2025-101066

**Published:** 2025-10-10

**Authors:** Eve Taylor, Ann McNeill, Harry Tattan-Birch, Tim Marczylo, Katherine East, Deborah Robson

**Affiliations:** 1 Department of Behavioural Science and Health, University College London, London, UK; 2 Addictions Department, Institute of Psychiatry, Psychology and Neuroscience, King’s College London, London, UK; 3 UK Health Security Agency (UKHSA), Didcot, UK; 4 Department of Primary Care and Public Health, University of Sussex, Brighton, UK

**Keywords:** Tobacco Use, TOXICOLOGY, Schizophrenia & psychotic disorders

## Abstract

**Abstract:**

**Objective:**

Smoking and vaping are especially prevalent among people with experience of psychosis (EoP), potentially increasing their toxicant exposure. Switching from tobacco smoking to vaping e-cigarettes reduces exposure to tobacco-related toxicants and likely associated diseases. We compared levels of nicotine and tobacco-related toxicant exposure among people with versus without EoP.

**Design:**

Cross-sectional study, secondary data analysis of Wave 5 (2018) of the Population Assessment of Tobacco and Health Study.

**Setting:**

Data collection took place in the USA at the home of participants.

**Participants:**

Data were from 5750 adults (aged >18 years) with and without EoP who smoked, vaped, did both or did neither. EoP was defined as ever being told by a health professional that you have schizophrenia, schizoaffective disorder, psychosis, a psychotic illness or psychotic episode.

**Primary outcome:**

Levels of urinary toxicants: nicotine metabolites, metals, volatile organic compounds (VOCs) and tobacco-specific nitrosamines (TSNAs) among people with and without EoP. Analyses were adjusted for demographics, cannabis use and past 30-day smoking/vaping status, and were repeated after stratifying by smoking /vaping status.

**Results:**

Of the 5750 participants, 6.3% (n=361) reported EoP, and 93.7% reported no EoP. Levels of nicotine and TSNA metabolites, cadmium, uranium and some VOCs were significantly higher among participants with EoP compared with those without. However, when smoking, vaping and cannabis use were taken into account, the associations of EoP with nicotine and TSNA metabolites, and most of the VOCs, were attenuated and no longer significant.

**Conclusion:**

Participants with EoP are exposed to more nicotine and tobacco-related toxicants than those without EoP, likely largely due to the high prevalence of smoking, vaping and cannabis use among this population.

**STRENGTHS AND LIMITATIONS OF THIS STUDY**

- This is some of the first research to focus on people with psychosis, a group who face a disproportionate burden of tobacco-related disease.
- The study has a large sample size from a population-based survey.
- Past 30-day use was used to define smoking/vaping status; this is sufficient to account for the washout of volatile organic compounds and NNN (N’-nitrosonornicotine), but likely not NNAL (4-(methylnitrosamino)-1-(3-pyridyl)-1-butanol) or metals.
- Smoking/vaping status was self-reported use status and may not be representative of their actual use, which would impact levels of toxicant exposure.

## Introduction

 People who experience psychosis face significant health inequalities, with a diagnosis of schizophrenia associated with a 10–20 year reduction in life expectancy.^1^ Although many factors contribute to this reduced life expectancy, differing rates of tobacco smoking are thought to be one of the leading contributors to health inequality between people with and without psychosis.^2^ In 2022, it was estimated that, in England, smoking prevalence was 15% among people overall and 43.1% among people with a history of psychosis.^3^ Similarly, high estimates of smoking prevalence among people with psychosis are seen in Australia (66.1%),^4^ Singapore (39.5%)^5^ and the USA (49.8%).^6^ The high prevalence of smoking among people with psychosis may be due to bidirectional causality, whereby smoking is associated with an early age of onset of psychosis and exacerbated psychosis symptoms,^7 8^ but also that people with psychosis are more likely to smoke for several reasons (eg, self-medication).^9^ Mendelian randomisation techniques have suggested that a genetic liability for lifetime smoking is a causal risk factor for schizophrenia.^10^

Smoking-related diseases are caused by exposure to numerous carcinogenic, cardiovascular and respiratory toxicants found in cigarettes and tobacco smoke. It is estimated that tobacco contains 93 harmful and potentially harmful constituents,^11^ many of which can be classed as: tobacco-specific nitrosamines (TSNAs), volatile organic compounds (VOCs), polycyclic aromatic hydrocarbons, metals and nicotine (table 1). Exposure to these toxicants can be estimated by measuring the levels of these toxicants, or their metabolites, in the bio-samples of people who smoke, commonly referred to as biomarkers of exposure. The term toxicant will be used throughout to refer to toxicants and their biomarkers of exposure.

**Table 1:** Urinary toxicants outcome variables

Toxicant	Metabolite		Known risks	Half-life^20 39^
	Full name	Abbreviation		
Nicotine	Cotinine		AD	16–18 hours
	3-hydroxycotinine	3-HC	AD	6–4 hours
Tobacco-specific nitrosamines				
NNK	4-(methylnitrosamino)-1-(3-pyridyl)-1-butanol	NNAL	CA	10.3 days
NNN	N’-nitrosonornicotine		CA	45 min
Metals				
Cadmium			CA, RT and RDT	13.6 years
Lead			CA, CT and RDT	1–2 months in soft tissue
Uranium			CA and RT	24 hours
Volatile organic compounds				
Acrolein	N-Acetyl-S-(3-hydroxypropyl)-L-cysteine	3-HPMA	CA, CT and RT	9 hours
	N-Acetyl-S-(2-carboxyethyl)-L-cysteine	CEMA		8 hours
Acrylamide	N-Acetyl-S-(2-carbamoylethyl)-L-cysteine	AAMA	CA	11–17 hours
Acrylonitrile	N-Acetyl-S-(2-cyanoethyl)-L-cysteine	CYMA	CA and RT	8 hours
Benzene	N-Acetyl-S-(phenyl)-L-cysteine	S-PMA	CA, CT and RDT	9 hours
1,3-Butadiene	N-Acetyl-S-(4-hydroxy-2-buten-1-yl)-L-cysteine	MHBMA3	CA, RT and RDT	5–9 hours
Crotonaldehyde	N-Acetyl-S-(3-hydroxypropyl-1-methyl)-L-cysteine	HPMMA	CA	5–9 hours
Ethylbenzene	Mandelic acid	MADA	CA	5 hours
Ethylene oxide	N-Acetyl-S-(2-hydroxyethyl)-L-cysteine	HEMA	CA, RT and RDT	-
Isoprene	*N*-acetyl-*S*-(4-hydroxy-2-methyl-2-buten-1-yl)-l-cysteine	IPM3	CA	1.25 hours
N,N-dimethylformamide	N-Acetyl-S-(N-methylcarbamoyl)-L-cysteine	AMCA	CA	23 hours
Propylene oxide	N-Acetyl-S-(2-hydroxypropyl)-L-cysteine	HPM2	CA and RT	–
Styrene	Phenylglyoxylic acid	PHGA	CA	8–10 hours
Xylene	3-Methylhippuric acid+4-Methylhippuric acid	34MH		34 hours

11

.AD, addictive; CA, carcinogenic; CT, cardiotoxic; RDT, reproductive toxicant; RT, respiratory toxic.

Exposure to these toxicants can be influenced by the patterns of use, such as cigarettes consumed per day,^12^ puff volume and puff duration.^13^ People with psychosis smoke tobacco cigarettes more heavily,^3^ have higher nicotine dependence^7^ and puff more intensively^14^ than people without psychosis. This may lead to people with psychosis being exposed to higher levels of toxicants, which would, in turn, contribute to the higher levels of tobacco disease seen in this community. Cannabis use is also substantially greater among people with psychosis than those without.^15^ Cannabis use, with and without tobacco, can also expose people to harmful toxicants. High levels of VOCs have been reported in the bio-samples of participants who smoke cannabis^16 17^ and are also reported in the aerosol of vaped cannabis.^18^ Metals are reported among people who smoke cannabis with and without tobacco.^19^ There is little evidence currently on the levels of tobacco toxicants that people with psychosis who smoke are exposed to, and if these are affected by patterns of use or co-use of cannabis.

High rates of vaping e-cigarettes are also reported among people with psychosis. It is estimated that, in England in 2022, vaping prevalence was 6.9% among people overall and 19.7% among people with a history of psychosis.^3^ E-cigarettes expose users to significantly fewer tobacco-related toxicants than smoking^20 21^ and can help some people quit smoking,^22^ including those with mental health conditions.^23^,^25^ However, similar to tobacco cigarettes, toxicant exposure from e-cigarettes can vary depending on behavioural or product characteristics such as heaviness of vaping and device or e-liquid type.^26 27^ Therefore, any differences in toxicant levels from vaping among people with psychosis compared with people without might be explained by the differences in vaping characteristics between these groups. There is, however, very little evidence from among people with psychosis who vape, especially research investigating the potential toxicant exposure from e-cigarettes. Thus, this paper aims to investigate levels of exposure to toxicants among people with and without experience of psychosis (EoP) who vape, smoke, do both concurrently or do neither.

## Methods

Methods and analysis plans were pre-registered on Open Science Framework https://osf.io/ctq9b/.

### Data source

Cross-sectional data were from Wave 5 (December 2018 to November 2019) of the US Population Assessment of Tobacco and Health (PATH) Study. The PATH Study is one of the largest national longitudinal cohort studies of tobacco use and health globally. It is run in collaboration between eight universities, the National Institute on Drug Abuse of the National Institutes of Health, the Center for Tobacco Products and the US Food and Drug Administration. Data collection for the PATH Study is conducted by Westat. Ethical approval for the analyses in this study was not required because this study involved secondary analysis of pre-existing data, in line with the policy of King’s College London. Participants provided in-person consent for both the survey and the bio-sample collection.

### Data collection

Researchers visited participants at their homes and used audio-computer assisted self-interviews to collect tobacco use behaviour, attitudes and beliefs and tobacco-related health outcomes of participants. Full-void urine samples were collected by participants at the time of the interview, though the provision of bio-samples was not a condition for inclusion. Adult respondents were paid $35 for their participation in the questionnaire, and an additional $25 for providing bio-samples.

### Participants

Persons aged ≥12 years and in the civilian non-institutionalised household population were eligible for participation in the PATH Study (See online supplemental file for sampling details). For this study, only data from participants aged >18 years and who provided urine samples were eligible for inclusion (n=7868). Of these, 1533 were excluded as they did not belong to one of the four pre-specified use groups (see Smoking/vaping variables section), and additional 2 were excluded for refusing to answer questions on sex. Participants were also excluded if they provided improbable responses (n=12), did not know responses (n=6) or refused responses (n=484) to how many days in the past month they used an e-cigarette/smoked a cigarette. An additional 83 were excluded because they had creatinine values outside the normal range of 10–370 mg/dL.^28^ This resulted in a total analytic sample of 5750.

For all analyses of nicotine and its metabolites, participants who had used nicotine replacement therapy (NRT) in the past 12 months (n=102) were excluded, as NRT use would lead to detection of elevated levels of nicotine and its metabolites in samples. For all other toxicant analyses, participants who had used NRT in the past 12 months were included because NRT would have little effect on levels of non-nicotine toxicants in samples.

### Measures

Outcomes of interest were urinary measures of tobacco-related toxicants. Toxicants of interest and their associated risks are outlined in table 1.

#### Predictor variables

Exact questionnaire wording is available in the pre-registration https://osf.io/ctq9b/.

#### Experience of psychosis

Participants were asked, “Has a doctor, therapist, or mental health professional ever told you that you have schizophrenia, schizoaffective disorder or psychosis?” Those who responded ‘no’ or ‘don’t know’ were then asked, “Has a doctor, therapist, or mental health professional ever told you that you had a psychotic illness or episode?” Responses were coded ‘Yes’, ‘Other’ (no, don’t know, refused). Responses were combined and any ‘yes’ response was coded as ‘EoP’. All other responses (including ‘don’t know’ n=7, and ‘refused’ n=13) were coded ‘No-EoP’. This coding is consistent with prior work using PATH data^29^ and can estimate similar population levels of psychosis as research using clinical records.^30^ See online supplemental material for full details.

### Smoking/vaping variables

#### Smoking/ vaping status

Smoking and vaping in the past 30 days was derived from multiple questions into four distinct groups. Participants who did not fulfil the criteria below were excluded:

- *Exclusively vaping:* vaped in the past 30 days and had not smoked cigarettes or used other tobacco products in the past 30 days.
- *Exclusively smoking*: smoked cigarettes in the past 30 days but had not vaped in the past 30 days (including those that had also used other tobacco products in the past 30 days).
- *Dual use (smoking and vaping):* smoked cigarettes and vaped in the past 30 days (including those that had also used other tobacco products in the past 30 days).
- *No-use:* not smoked or vaped in the past 12 months. A stricter criteria of 12 months was chosen for no-use as 30 days would not be sufficient for the washout of some toxicants and residual levels would still be detected (table 1). Participants who had used other tobacco products in the past 30 days were also excluded.

#### Vaping, smoking and other product use characteristic variables

- Other tobacco products: past 30-day use of cigarillo or filtered cigar, hookah, snus or other smokeless tobacco (‘yes’, ‘no’).
- Past 12-month use of NRT (‘yes’ or ‘no’).
- Past 30-day use of cannabis (defined in the questionnaire as marijuana, hash, THC (delta-9-tetrahydrocannabinol), grass, pot or weed). Responses were coded ‘yes’ or ‘other (no, refused)’.
- Smoking characteristics: Days smoked in the past 30 days (linear variable). Type of cigarette smoked (‘exclusively roll-your-own’, ‘exclusively manufactured’, ‘roll-your-own and manufactured’, ‘don’t know’). Menthol cigarette use (‘yes’, ‘other (no, don’t know)’. Heaviness of Smoking Index (HSI; High’, ‘Medium’, ‘Low’) in accordance with Heatherton *et al*^31^ and only among people who smoked daily.
- Vaping characteristics: Days vaped in the past 30 days (linear variable). Type of vape (‘disposable’, ‘pod’, ‘tank’, ‘other’). Vape usually contains nicotine (‘yes’ or ‘no’). Usual nicotine content (‘6 mg (0.6%) or less’, ‘7 mg (0.7%) to 12 mg (1.2%)’, ‘13 mg (1.3%) to 24 mg (2.4%)’, ‘25 mg (2.5%) or more’), ‘don’t know’. Usual flavour vaped (‘tobacco’, ‘mint’, ‘fruit’, ‘other’).
- Demographic variables: age (linear variable), sex (‘male’, ‘female’) and ethnicity (coded ‘White’, ‘Black’ and ‘Other ethnicity’ due to small sample sizes).

### Sample handling and analysis

Bio-samples were analysed at the CDC National Centre for Environmental Health, Division of Laboratory Sciences for analysis.^32^ See Online supplemental file for details.

### Data analysis

Analyses were pre-registered on the Open Science Framework https://osf.io/ctq9b/.

Toxicants with values below the Limit of Detection (LOD) were given a value of LOD/√2 as is recommended to account for inaccuracies of levels detected below LOD.^33^ Due to non-standard distributions of toxicant data, geometric means and 95% CIs were reported, and log-transformed values were used for regression analyses. To account for variations in hydration and renal function between the participants, toxicant levels were corrected for creatinine by reporting the level of toxicant in the urine/the level of creatinine (dL) in the urine.

Descriptive statistics were used to report the prevalence of smoking/vaping, as well as the frequency of use, product characteristics, other tobacco, nicotine and cannabis use, and demographic variables among people with and without EoP.

Linear regressions were used to examine levels of toxicants by EoP, adjusted for age, sex and ethnicity (model 1), smoking and vaping status, age, sex and ethnicity (model 2), and past 30-day cannabis use, smoking and vaping status, age, sex and ethnicity (model 3). Finally, interaction effects were then introduced to model 3, to explore differences in levels of toxicants by smoking and vaping status within and between EoP.

#### Deviation from pre-registration

As cannabis use may affect exposure to levels of toxicants,^17^ interactions were added to models that adjust for cannabis (model 3) and not just smoking and vaping (model 2), as had been originally stated in the pre-registered data analysis plan.

To investigate the effect of device and use characteristics on toxicant exposure, exploratory analyses were also conducted for toxicants where significant interactions between smoking and vaping status and EoP were detected. These analyses used logistic regression to examine associations between toxicant levels and HSI scores among participants who smoked, and nicotine concentration and vaping device type among participants who vaped. All analyses were adjusted for age, sex, ethnicity and past 30-day cannabis use.

### Patient and public involvement

As this was a secondary data analysis, it was not appropriate or possible to involve patients or the public in the design, conduct, reporting or dissemination plans of our research.

## Results

### Participant characteristics

Across the analytical sample, 6.3% of participants reported having an EoP (table 2). The majority of the sample exclusively smoked (48.8%), followed by neither vaping nor smoking (32.6%), dual using (13.5%) and, finally, exclusively vaping (5.2%). Due to the sample selection criteria, these proportions are not representative of population levels of smoking and vaping.

**Table 2:** Participant characteristics (n=5750)

	Total		EoP		No EoP	
	%	N	%	N	%	N
		5750	6.3	361	93.7	5389
Smoking/vaping in the past 30-days						
Exclusively vaped (vape)	5.2	297	5.0	18	5.2	279
Exclusively smoked (smoke)	48.8	2803	59.6	215	48.0	2588
Both smoked and vaped (dual)	13.5	775	21.3	77	13.0	698
Neither vape nor smoke	32.6	1875	14.1	51	33.9	1824
Age	M=42.0	SD=15.0	M=42.0	SD=13.0	M=42.0	SD=15.1
Ethnicity						
White	72.2	4152	65.9	238	72.6	3914
Black	17.7	1020	23.6	85	17.4	935
Other ethnicity	10.1	578	10.5	38	10.0	540
Sex						
Male	47.4	2727	42.1	152	47.8	2575
Female	52.6	3023	57.9	209	52.2	2814
Past 30-day cannabis use	25.3	1456	37.4	135	24.5	1321
Past 12-month nicotine replacement therapy use	1.8	102	7.2	26	1.4	76

Characteristics are unweighted.

Among all participants who exclusively vaped, 82.5% had previously smoked, with 33% having smoked in the past 12 months. Among participants who neither vaped nor smoked, 27.6% had previously smoked (table 3).

**Table 3:** Participant smoking and vaping characteristics

	Vape (n=297)	Smoke (n=2803)	Dual (n=775)
Other tobacco use			
Cigarillo	–	11.7 (324)	24.3 (188)
Filtered cigar	–	5.9 (165)	13.7 (106)
Cigar	–	17.0 (477)	32.9 (255)
Pipe	–	1.7 (48)	6.5 (50)
Hookah	–	2.3 (64)	9.8 (76)
Snus	–	1.5 (42)	5.3 (41)
Smokeless tobacco	–	4.3 (121)	8.0 (62)
Smoking characteristics			
Days smoked in the past 30 days	–	M=26.0, SD=8.8	M=22.1, SD=11.3
Currently smoking daily	–	77.4 (2169)	59.6 (462)
Smoke menthol	–	43.9 (1229)	49.7 (385)
Heaviness of Smoking Index			
Low	–	20.5 (444)	21.4 (98)
Moderate	–	71.6 (1551)	73.0 (335)
High	–	7.9 (170)	5.7 (26)
Vaping characteristics			
Days vaped in the past 30 days	M=23.4, SD=11.4	–	M=14.5, SD=12.2
Currently vaping daily	70.4 (209)	–	27.7 (215)
Device type*			
Disposable	3.0 (9)	–	11.1 (86)
Pod	30.0 (89)	–	34.1 (264)
Tank	66.3 (197)	–	53.9 (418)
Nicotine concentration †			
0 mg	13.5 (40)	–	11.5 (89)
1–6 mg	47.8 (142)	–	35.0 (271)
7–12 mg	3.4 (10)	–	7.1 (55)
13–24 mg	7.7 (23)	–	7.4 (57)
25^+^mg	14.8 (44)	–	10.1 (78)
Flavour vaped ‡			
Tobacco	14.1 (42)	–	22.5 (174)
Mint	21.2 (63)	–	24.4 (189)
Fruit	44.1 (131)	–	43.6 (338)
Other	34.0 (101)	–	27.7 (215)

* n=9 reported did not know or refused to answer device type question.

† n=263 reported did not know or refused to answer nicotine concentration vaped question.

‡ Participants could choose multiple flavours, groups are not exclusive.

Among participants with EoP, 80.8% who exclusively smoked were smoking daily, and 64.9% who dual used were smoking daily. Among participants without EoP, 77.1% who exclusively smoked were smoking daily, and 59% who dual used were smoking daily (online supplemental table S1). Among participants with EoP, 13.2% who smoked and 12% who dual used had high HSI scores. Among participants without EoP, 7.4% who smoked and 4.9% who dual used had high HSI scores (online supplemental table S1).

Among those who exclusively vaped, most participants with EoP (83.3%) and without EoP (69.5%) vaped daily. Just over a quarter of participants who dual used vaped daily (EoP 24.7%, no-EoP 28.1%; online supplemental table S2).

### Toxicant results

Findings from the interaction analyses of comparisons of toxicant levels within participants with EoP and within participants without EoP are outlined in online supplemental table S10.

### Nicotine metabolites

Levels of cotinine and 3-hydroxycotinine (3-HC) were significantly higher among participants with EoP compared with participants without. When models were adjusted for smoking/vaping status, the effect of EoP lost significance (online supplemental table S3).

#### Interactions

**Figure 1:**
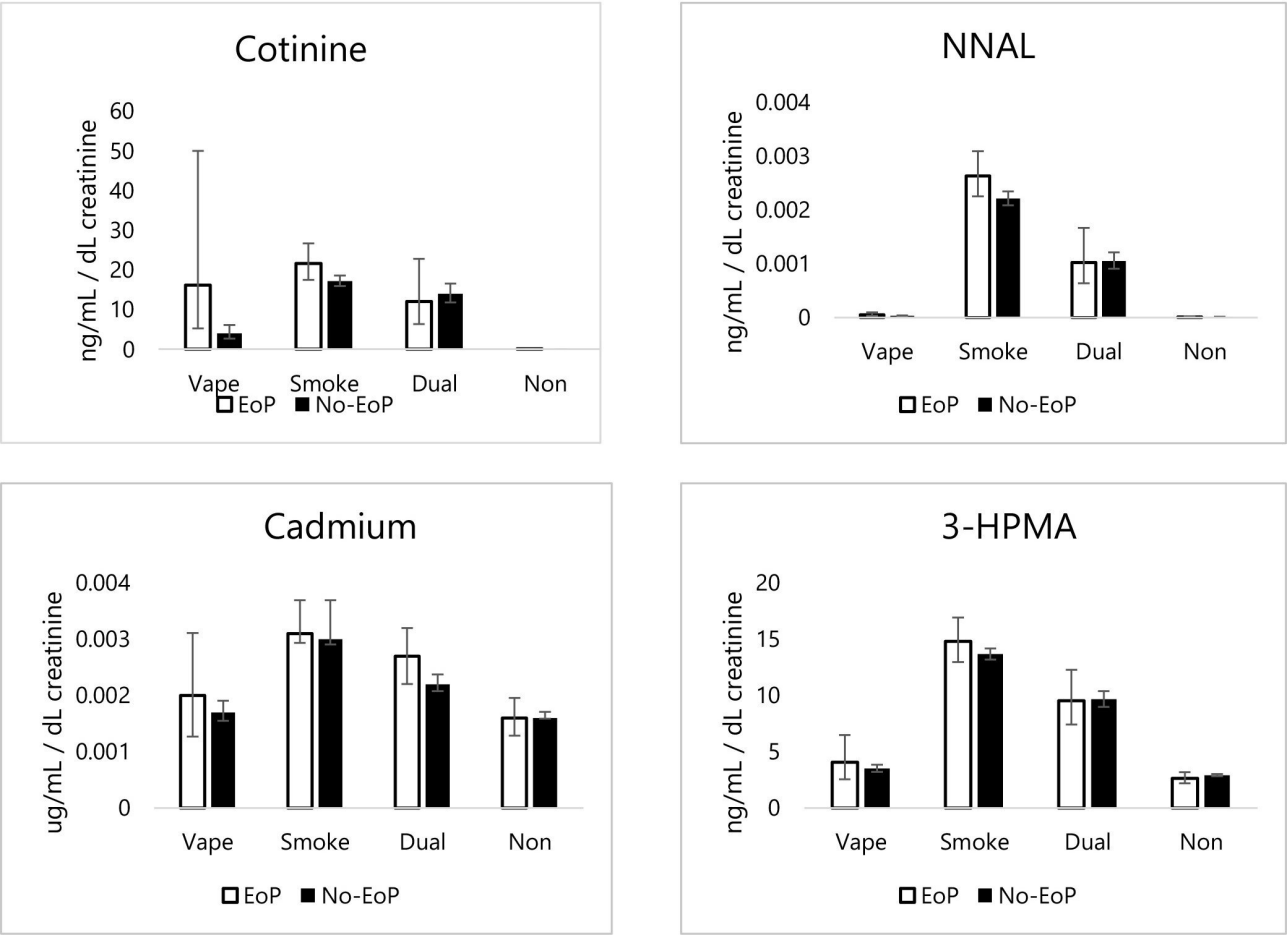
Geometric mean levels of cotinine, NNAL, cadmium and 3-HPMA by EoP and vaping/smoking status. EoP, experience of psychosis; NNAL, 4-(methylnitrosamino)-1-(3-pyridyl)-1-butanol; 3-HPMA, N-Acetyl-S-(3-hydroxypropyl)-L-cysteine.

When interactions between smoking/vaping status and EoP were explored, cotinine and 3-HC levels were significantly higher among participants with EoP who only vaped compared with participants without EoP who only vaped (cotinine, geometric mean (GM) EoP=16.18 vs GM no-EoP=4.03, adjusted odds ratio (AOR)=−1.38, 95% CI=−2.56 to 0.19, p=0.023; 3-HC, GM EoP=29.36 vs GM no-EoP=6.98, AOR=−1.37, 95% CI=−2.56 to 0.18, p=0.024; figure 1).

When the interactions were investigated further among participants who vaped, associations between EoP and levels of cotinine and 3-HC lost significance when adjusted for device type or nicotine concentration. Participants who vaped disposable products, but not pod products, had significantly lower levels of cotinine and 3-HC compared with tank products. Participants who vaped 0 mg nicotine liquids, but not 1–12 mg, had significantly lower levels of cotinine and 3-HC than those who vaped 13 mg or over (online supplemental table S11).

### Tobacco-specific nitrosamines

In model 1, levels of 4-(methylnitrosamino)-1-(3-pyridyl)-1-butanol (NNAL) and N’-nitrosonornicotine (NNN) were significantly higher among participants with EoP compared with participants without. When models were adjusted for smoking/vaping status in model 2, there was no longer a significant effect (online supplemental table S3).

#### Interactions

When interactions between smoking/vaping status and EoP were explored, there were no significant differences found between people with and without EoP (figure 1).

### Metals

In model 1, levels of cadmium and uranium, but not lead, were significantly higher among participants with EoP compared with participants without. When vaping and smoking status were controlled for in model 2, differences in cadmium and uranium among participants with and without EoP remained significant. For model 3, differences in cadmium, but not uranium, among participants with and without EoP remained significant when adjusted for cannabis use (online supplemental table S4).

#### Interactions

When interactions between smoking/vaping status and EoP were explored, differences in levels of cadmium among people with and without EoP who smoked had borderline significance (AOR=−0.09, 95% CI=−0.18 to -0.00008, p=0.050; figure 1). The association became non-significant when analyses were adjusted for HSI, with cadmium levels significantly higher among people with high or moderate HSI scores compared with low (online supplemental table S13).

### Volatile organic compounds

Levels of AMCA, CEMA and PMA did not differ significantly between participants with and without EoP (model 1), between vaping/smoking status (model 2), or who had used cannabis in the past 30 days (model 3; online supplemental tables S5–S9).

Levels of 34MH, 3-HPMA, MHB3, HPMM, HPM2, AAMA HEMA, PHGA, IPM3, MADA and CYMA were significantly higher among participants with EoP than without in model 1. When models were adjusted for smoking/vaping status, the effect of EoP only remained significant for HPMM, IPM3 and MADA (model 2). When adjusting for cannabis use (model 3), the effect of EoP only remained significant for IPM3 and MADA (online supplemental tables S5–S9).

#### Interactions

When interactions between smoking/vaping status and EoP were explored, levels of 34MH were significantly higher among participants with EoP who vaped compared with participants without EoP who vaped (GM EoP =2.42 vs GM no-EoP=1.43, AOR=−0.50, 95% CI=−0.93 to 0.07, p=0.022). When this was investigated further, among people who vaped, associations between EoP and levels of 34MH remained significant when adjusted for device type or nicotine concentration, and there were no significant associations between 34MH and device type or nicotine concentration (online supplemental table S12).

Levels of AAMA and HPM2 were significantly lower among participants with EoP who vaped compared with participants without EoP who vaped (AAMA, EoP=0.42 vs GM no-EoP=0.62, AOR=0.46, 95% CI=0.14 to 0.78, p=0.005; HPM2, GM EoP=0.25 vs GM no-EoP=0.37, AOR=0.40, 95% CI=0.02 to 0.78, p=0.039). Among people who vaped, associations between EoP and levels of AAMA and HPM2 remained significant when adjusted for device type or nicotine concentration. People who used disposable vapes had significantly higher levels of AAMA compared with people who used tank devices (online supplemental table S12).

Levels of HPMM, IPM3 and MADA were significantly higher among participants with EoP who smoked compared with participants without EoP who smoked (HPMM, GM EoP=13.44 vs GM no-EoP=11.91, AOR=−0.09, 95% CI=−0.17 to 0.01, p=0.024; IPM3, GM EoP=0.40 vs GM no-EoP=0.35, AOR=−0.14, 95% CI=−0.30 to 0.01, p=0.045; MADA, GM EoP=3.43 vs GM no-EoP=3.14, AOR=−0.10, 95% CI=−0.17 to 0.02, p=0.017).

Among people who smoked, associations between EoP and levels of HPMM, IPM3 and MADA became non-significant when analyses were adjusted for HSI. Levels of all three toxicants were significantly higher among people with high or moderate HSI scores compared with low (online supplemental table S13).

### Associations between toxicant exposure and cannabis

In model 3, when adjusting for EoP and smoking and vaping status, levels of lead, uranium, 34MH, 3-HPMA, AAMA, CYMA, HPM2, HEMA, HPMM, IPM3, MHB3, MADA and PHGA were significantly greater among people who used cannabis compared with people who did not (online supplemental table S13).

## Discussion

Overall, nicotine and TSNA metabolites, cadmium, uranium and some VOCs were significantly higher among participants with EoP compared with those without. However, when smoking and vaping status were taken into account, the association of EoP with nicotine and TSNA metabolites and most of the VOCs was no longer significant. For cadmium, uranium and three VOCs (HPMM, IPM3 and MADA), the effect of EoP remained significant when smoking and vaping status were taken into effect, although when controlling for cannabis use, uranium and HPMM were no longer significant. When exploring interactions between smoking/vaping status and EoP, higher nicotine metabolite levels among people with EoP who vaped seemed to be accounted for by the devices used; for example, disposables were associated with significantly lower levels of nicotine metabolites. For three VOCs (34MH, AAMA and HPM2), levels were lower among people with EoP who vaped than those without, and these remained significant when adjusting for device type or nicotine concentration. For three other VOCs (HPMM, IPM3 and MADA), levels were higher among participants with EoP who smoked compared with those without, but these differences seemed to be accounted for by higher dependence on cigarettes among those with EoP. The findings overall suggest that the significantly higher levels of toxicant exposure among participants with EoP are largely due to a higher prevalence of smoking, vaping and cannabis use as well as differences in dependence on smoking and vaping devices/nicotine concentrations used for nicotine metabolites. Across all participants, levels of TSNAs, metals and most VOCs were significantly lower among those who vaped compared with those who smoked or dual used.

The higher consumption of nicotine among people with EoP may be linked to higher levels of dependence among this group. People with psychosis experience higher levels of tobacco dependence than people without,^7^ therefore they likely require higher levels of nicotine to suppress withdrawal and craving, and remain abstinent from cigarettes. The significant effects of device type and AAMA may be due to differences in e-liquids, as tank devices are typically used with higher vegetable glycerin content e-liquids, which can produce lower levels of VOCs than 100% propylene glycol liquids used in other models.^34^ Findings should, however, be interpreted with caution due to the small sample size of people with EoP who exclusively vaped.

Past 30-day cannabis use was substantially higher among participants with EoP than participants without, which is in line with previous research from the USA.^15^ Past 30-day cannabis use was associated with levels of lead, uranium and most VOCs. These findings are consistent with previous research reporting high levels of VOCs in bio-samples of participants who smoke cannabis.^16 17^ Associations between EoP, uranium and the VOC HPMM lost significance when adjusting for cannabis use. This suggests that greater cannabis use among people with EoP contributes to higher exposure to these toxicants among this population. Cannabis use was not found to moderate levels of nicotine metabolites or TSNAs. This is consistent with findings of very low levels of NNAL and total nicotine equivalents among participants who exclusively smoke cannabis without tobacco.^35^ However, the present study used data from the USA where mixing cannabis with tobacco is rare^36^ and so findings may not generalise to other countries where mixing cannabis with tobacco is more common (eg, England).

Across all participants, levels of TSNAs, metals and most VOCs were significantly lower among people who vaped compared with those who smoked or dual used, which is consistent with previous research.^20 21^ Levels of metals and some VOCs were greater among people who vaped compared with those who neither vaped nor smoked; however, so were levels of TSNAs and the VOC CYMA, both of which indicate recent tobacco exposure among people who had vaped.^37^ This supports the harm reduction potential of e-cigarettes as a smoking cessation tool for people with EoP, particularly as we found that they are exposed to significantly higher levels of cadmium and some VOCs due to their heaviness of tobacco smoking than people who smoke without EoP. Greater rates of cannabis consumption also contributed to higher levels of toxicant exposure. These findings emphasise the need for targeted stop-smoking support and cannabis harm reduction interventions for this population to reduce toxicant exposure and subsequent disease.

There are limitations to this research. First, definitions of exclusive vaping, smoking and dual use may not adequately account for washout periods. Exclusive past 30-day use should be sufficient to account for the washout of VOCs and NNN from other products; however, 6 months would have been preferable to ensure a complete washout from prior use for NNAL. Second, PATH methods do not include CO (carbon monoxide) readings to bio-verify the tobacco use status of participants; thus, bio-verification could not be used as a condition for inclusion. Therefore, the self-reported use status of participants may not be representative of their actual use, which would impact levels of toxicant exposure. Third, data were from the USA, so may not be as applicable to other countries (eg, in England where there is a limit of 20 mg/mL on nicotine content in vapes, or in Australia where nicotine-containing vapes are illegal without prescription).^38^ Finally, the number of participants with a history of psychosis in the sample was relatively small (n=361), which may limit the precision of estimates for this group.

### Conclusion

Overall, participants with EoP had higher levels of nicotine and tobacco-related toxicants in urine samples than participants without EoP. These differences attenuated and became non-significant after adjustment for smoking/vaping and cannabis use, suggesting that this increased exposure is largely due to the high prevalence of these behaviours among people with EoP. Nicotine and some VOCs differed between participants with EoP who vaped compared with participants without EoP who vaped, potentially due to differences in the way vaping products are used between these groups. These findings identify elevated exposure to disease-related toxicants among participants with EoP, a likely causal factor in the high levels of morbidity and mortality among this population.

## Supplementary material

10.1136/bmjopen-2025-101066 online supplemental file 1

## Data Availability

Data may be obtained from a third party and are not publicly available.
