# Supplementary material for: Tobacco-related toxicant exposure among people with and without experience of psychosis: findings from the US Population Assessment of Tobacco and Health study
Source: BMJ Open. 2025 Oct 10;15(10):e101066. doi: 10.1136/bmjopen-2025-101066 (PMC12517016; doi:10.1136/bmjopen-2025-101066)
Supplement: online supplemental file 1 [file bmjopen-15-10-s001.docx]

## Supplementary methods

Sampling:

The initial sample was recruited in Wave 1 (2015) using a stratified area-probability sampling design. Over 150,000 US mailing addresses were sampled, leading to a Wave 1 adult (18+years) sample of 32,320. Of the original Wave 1 cohort, 7,868 (24.3%) provided urine samples and questionnaire data during Wave 5 recontact. Additional details on sampling methods are provided elsewhere (Inter-university Consortium for Political and Social Research, 2023).

### Sample handling and analysis:

Bio-samples were placed in temperature-controlled containers by field researchers and shipped to the CDC National Center for Environmental Health, Division of Laboratory Sciences for analysis (Sosnoff & Pirkle, 2016). Analysis was conducted by the CDC in accordance with their pre-defined methods. In brief, nicotine, cotinine and 3-HC were quantified using liquid chromatography/atmospheric pressure chemical ionization tandem mass spectrometry. Metals were quantified using inductively coupled plasma mass spectrometry in combination with dynamic reaction cell technology. TSNAs were quantified using isotope dilution high performance liquid chromatography/atmospheric pressure ionization tandem mass spectrometry (HPLC-MS/MS). VOCs were quantified using ultra performance liquid chromatography coupled with electrospray ionization tandem mass spectrometry. Quality assessment was analysed through the inclusion of blinded replicate samples and quality control samples randomly added to sample shipments. Full details of analytical methods are available elsewhere (Sosnoff & Pirkle, 2016).

### Supplementary results

### Nicotine metabolites (Table S3)

##### Differences within participants with EoP

Compared to participants with EoP who only vaped, levels of cotinine and 3-HC were significantly lower among participants with EoP who neither vaped nor smoked, but not significantly different to participants who only smoked or dual used (Figure 1a, Table S10).

##### Differences within participants without EoP

Compared to participants without EoP who only vaped, levels of cotinine and 3-HC were significantly lower among participants without EoP who neither vaped nor smoked, but significantly higher among participants who only smoked or dual used (Figure 1a, Table S10).

#### TSNAs (Table S3)

##### Differences within participants with EoP

Among participants with EoP who only vaped, levels of NNAL and NNN were significantly lower than participants who only smoked or dual used. Levels of NNAL, but not NNN were significantly higher than those who neither vaped nor smoked compared to vaped (Figure 1b, Table S10). This suggests historic tobacco exposure, given the much shorter half-life for NNN compared to NNAL.

Differences within participants without EoP

Among participants without EoP who vaped, levels of NNAL and NNN were significantly lower than participants who smoked or dual used, but higher than those who neither vaped nor smoked (Figure, 1b, Table S10).

### Metals (Table S4)

##### Differences within participants with EoP

Compared to participants with EoP who vaped, levels of cadmium were significantly higher among participants with EoP who smoked or dual used, but not significantly different to participants who neither smoked nor vaped. There were no significant differences in levels of lead or uranium (Figure 1c, Table S10).

##### Differences within participants without EoP

Compared to participants without EoP who only vaped, levels of lead and cadmium were significantly higher among participants without EoP who smoked or dual used, but significantly lower among participants who neither smoked nor vaped. Levels of uranium were significantly higher among participants who smoked, lower among participants who neither vaped nor smoked, and not significantly different to dual use, compared to participants without EoP who vaped (Figure 1c, Table S10)

### VOCs (Tables S5-S9)

###### Differences within participants with EoP

Compared to participants with EoP who vaped, most VOCs, except for AMCA, CEMA or PMA, were significantly higher among participants with EoP who smoked or dual used (Table S10). Except for 34MH, there were no significant differences in VOC levels between participants who vaped and those who neither vaped nor smoked.

###### Differences within participants without EoP

Compared to participants without EoP who vaped, levels of all VOCs, except for AMCA, CEMA or PMA, were significantly higher among participants without EoP who smoked or dual used. Levels of 3-HPMA, CYMA, and MADA were higher among participants who vaped than those who neither vaped nor smoked (Figure 1d, Table S10).

| Table S1: Participant smoking characteristics by Evidence of Psychosis (EoP) | | | | |
| --- | --- | --- | --- | --- |
|  | Smoke only | | Dual use | |
|  | EoP (n=215) | No-EoP (n=2588) | EoP (n=77) | No-EoP (n=698) |
| **Other tobacco use**^a^ |  |  |  |  |
| *Cigarillo* | 33.8(26) | 11.4(294) | 33.7(26) | 23.2(162) |
| *Filtered cigar* | 13.1(28) | 5.3(137) | 18.2(14) | 13.2(92) |
| *Cigar* | 21.9(47) | 16.6(430) | 40.3(31) | 32.1(224) |
| *Pipe* | 4.2(9) | 1.5(39) | 20.8(16) | 4.9(34) |
| *Hookah* | 2.8(6) | 2.2(58) | 15.6(12) | 9.2(64) |
| *Snus* | 2.3(5) | 1.4(37) | 11.7(9) | 4.6(32) |
| *Smokeless tobacco* | 4.7(10) | 4.3(111) | 16.9(13) | 7.0(49) |
| **Smoking characteristics** |  |  |  |  |
| Days smoked in the past 30 | M=27.0 SD=7.8 | M=26.0 SD=8.9 | M=22.9 SD=11.5 | M=22.0 SD=11.3 |
| Currently smoking daily | 80.8(174) | 77.1(1995) | 64.9(50) | 59.0(412) |
| Smoke Menthol | 55.4(119) | 42.9(1110) | 57.1(44) | 49.7(341) |
| **HSI** |  |  |  |  |
| *Low* | 13.2(23) | 21.2(421) | 20.0(10) | 21.5(88) |
| *Moderate* | 73.6(128) | 71.5(1423) | 68.0(34) | 73.6(301) |
| *High* | 13.2(23) | 7.4(147) | 12.0(6) | 4.9(20) |
| ^a^ Participants could report use of multiple other tobacco products | | | | |

| Table S2: Participant vaping characteristics by EoP | | | | |
| --- | --- | --- | --- | --- |
|  | Vape only | | Dual use | |
|  | EoP n=18 | No-EoP n=279 | EoP n=77 | No-EoP n=698 |
|  | %(n) | %(n) | %(n) | %(n) |
| **Vaping characteristics** |  |  |  |  |
| Days vaped in the past 30 days | M=30 SD=0 | M=23 SDS=11.6 | M=11.9 SD=12.1 | M=14.9 SD=12.2 |
| Currently vape daily | 83.3(15) | 69.5(194) | 24.7(19) | 28.1(196) |
| **Device type** ^a^ |  |  |  |  |
| *Disposable* | 0 | 3.2(9) | 22.1(17) | 9.9(69) |
| *Pod* | 16.7(3) | 30.8(86) | 29.9(23) | 34.5(241) |
| *Tank* | 83.3(15) | 65.2(182) | 46.8(36) | 54.7(382) |
| **Nicotine concentration** ^b^ |  |  |  |  |
| *0-12mg* | 66.7(12) | 64.5(172) | 50.7(39) | 53.9(376) |
| *13+mg* | 27.7(5) | 22.2(71) | 14.3(11) | 17.7(124) |
| **Flavour vaped** ^c^ |  |  |  |  |
| *Tobacco* | 11.1(2) | 24.7(19) | 14.34(40) | 22.2(155) |
| *Mint* | 27.8(5) | 20.8(58) | 29.9(23) | 23.8(166) |
| *Fruit* | 44.4(8) | 44.1(123) | 44.2(34) | 43.6(304) |
| *Other* | 33.3(6) | 34.1(95) | 29.9(23) | 27.5(192) |
| ^a^n=9 reported don’t know or refused to device type question  ^b^n=263 reported don’t know or refused to nicotine question  ^c^ Participants could choose multiple flavours, groups are not exclusive | | | | |

| Table S3: associations between biomarker levels, EoP, Vaping/smoking and cannabis (N=5750) | | | | | | | | | | | | |
| --- | --- | --- | --- | --- | --- | --- | --- | --- | --- | --- | --- | --- |
|  | Cotinine* ng/ml (n=5646) | | | 3-HC* ng/ml (n=5644) | | | NNAL* ng/ml (n=5718) | | | NNN* ng/ml (n=5660) | | |
|  | GM(95% CI) | Beta (95% CI) | p | GM(95% CI) | Beta (95% CI) | p | GM(95% CI) | Beta (95% CI) | p | GM(95% CI) | Beta (95% CI) | p |
| Total | 1065.5  (949.34-1195.88) |  |  |  |  |  | 0.31(0.28-0.33) |  |  | 0.04  (0.04-0.05) |  |  |
| **Model 1** |  |  |  |  |  |  |  |  |  |  |  |  |
| EoP | 5570.2  (3838.7-8082.7) | -1.82  (-2.30- -1.33) | <.001 | 10.0  (6.93-14.52) | -1.81  (-2.29- -1.32) | <.001 | 0.86(0.67-1.1) | -1.13  (-1.43 - -0.83) | <.001 | 0.06  (0.05-0.06) | -0.28  (-0.40- -0.16) | <.001 |
|  |  |  |  |  |  |  |  |  |  |  |  |  |
| **Model 2** |  |  |  |  |  |  |  |  |  |  |  |  |
| EoP |  |  |  |  |  |  |  |  |  |  |  |  |
| No-EoP |  | -0.21(-0.46-0.04) | .106 |  | -0.22(-0.47-0.03) | .090 |  | -0.15(-0.31-0.01) | .075 |  | -0.05(-0.15-0.05) | .349 |
|  |  |  |  |  |  |  |  |  |  |  |  |  |
| Vape | 4346.5  (2919.5-6470.9) |  |  | 7.5  (5.0-11.27) |  |  | 0.03(0.03-0.04) |  |  | 0.03  (0.02-0.03) |  |  |
| Smoke | 17512.7  (16263-18858.4) | 1.31(1.03-1.59) | **<.001** | 30.6  (28.4-32.9) | 1.25(0.97-1.54) | **<.001** | 2.24(2.12-2.37) | 4.1(3.92-4.29) | **<.001** | 0.07  (0.07-0.08) | 0.98  (0.87-1.1) | **<.001** |
| Dual | 13811.4  (11729.5-16262.8) | 1.16(0.84-1.47) | **<.001** | 22.6  (19.22-26.68) | 1.09(0.77-1.4) | **<.001** | 1.05(0.91-1.2) | 3.47(3.27-3.68) | **<.001** | 0.06  (0.05-0.06) | 0.79  (0.66-0.92) | **<.001** |
| Non-use | 4.9(4.3-5.5) | -6.83(-7.12--6.55) | **<.001** | 0.01  (0.008-0.01) | -6.8(-7.08--6.51) | **<.001** | 0.01(0.01-0.01) | -0.99(-1.18--0.80) | **<.001** | 0.02  (0.02-0.02) | -0.22  (-0.33--0.1) | **<.001** |
| **Model 3** |  |  |  |  |  |  |  |  |  |  |  |  |
| EoP |  |  |  |  |  |  |  |  |  |  |  |  |
| No-EoP |  | -0.2(-0.46-0.05) | .115 |  | -0.21(-0.47-0.04) | .102 |  | -0.14(-0.31-0.02) | .087 |  | -0.05(-0.16-0.05) | .317 |
|  |  |  |  |  |  |  |  |  |  |  |  |  |
| Vape |  |  |  |  |  |  |  |  |  |  |  |  |
| Smoke |  | 1.31(1.03-1.59) | **<.001** |  | 1.26(0.97-1.54) | **<.001** |  | 4.1(3.92-4.29) | **<.001** |  | 0.98(0.87-1.1) | **<.001** |
| Dual |  | 1.15(0.84-1.47) | **<.001** |  | 1.08(0.76-1.39) | **<.001** |  | 3.46(3.26-3.67) | **<.001** |  | 0.79(0.67-0.92) | **<.001** |
| Non-use |  | -6.82(-7.11--6.53) | **<.001** |  | -6.78(-7.07--6.49) | **<.001** |  | -0.98(-1.17--0.79) | **<.001** |  | -0.23(-0.34--0.11) | **<.001** |
|  |  |  |  |  |  |  |  |  |  |  |  |  |
| Cannabis use | 4898.6  (4134.1-5804.5) |  |  | 8.6  (7.2-10.2) |  |  | 0.68(0.6-0.77) |  |  | 0.05  (0.05-0.05) |  |  |
| No-cannabis use | 639.7  (556.2-735.8) | -0.07(-0.21-0.08) | 0.371 | 1.2(1.00-1.32) | -0.09(-0.24-0.05) | .215 | 0.68(0.6-0.77) | -0.07(-0.17-0.02) | .126 | 0.04(0.04-0.04) | 0.04(-0.02-0.1) | .144 |
| Biomarker levels were adjusted for mg/dL creatinine  All analyses were adjusted for age, sex and ethnicity.  *102 participants were removed who had used NRT in the past 12 months.  BoEs are presented by group and then ordered alphabetically. | | | | | | | | | | | | |

| Table S4: associations between biomarker levels, EoP, Vaping/smoking and cannabis pg/ml creatinine (N=5750) | | | | | | | | | |
| --- | --- | --- | --- | --- | --- | --- | --- | --- | --- |
|  | Cadmium ug/l (n=5749) | | | Lead ug/l (n=5749) | | | Uranium ug/l (n=5749) | | |
|  | GM(95% CI) | Beta (95% CI) | p | GM(95% CI) | Beta (95% CI) | p | GM(95% CI) | Beta (95% CI) | p |
| Total | 2.3(2.3-2.4) |  |  | 3.5(3.4-3.5) |  |  | 0.07(0.07-0.07) |  |  |
| **Model 1** |  |  |  |  |  |  |  |  |  |
| EoP | 2.8(2.5-3) |  |  | 3.6(3.4-3.9) |  |  | 0.08(0.07-0.09) |  |  |
| No-EoP | 2.3(2.2-2.3) | -0.2(-0.27--0.12) | **<.001** | 3.5(3.4-3.5) | -0.07(-0.14-0) | .052 | 0.07(0.07-0.07) | -0.16(-0.27--0.06) | **.002** |
| **Model 2** |  |  |  |  |  |  |  |  |  |
| EoP |  |  |  |  |  |  |  |  |  |
| No-EoP |  | -0.09(-0.16--0.02) | **.011** |  | 0(-0.07-0.07) | .958 |  | -0.11(-0.21-0) | **.046** |
|  |  |  |  |  |  |  |  |  |  |
| Vape | 1.7(1.6-1.9) |  |  | 3(2.8-3.3) |  |  | 0.07(0.06-0.07) |  |  |
| Smoke | 3(2.9-3.1) | 0.28(0.2-0.36) | **<.001** | 4.1(4-4.2) | 0.19(0.11-0.27) | **<.001** | 0.08(0.07-0.08) | 0.16(0.04-0.27) | **.009** |
| Dual | 2.3(2.1-2.4) | 0.25(0.17-0.34) | **<.001** | 3.5(3.4-3.7) | 0.17(0.08-0.26) | **<.001** | 0.07(0.07-0.08) | 0.09(-0.04-0.22) | .179 |
| Non-use | 1.6(1.6-1.7) | -0.26(-0.34--0.19) | **<.001** | 2.7(2.6-2.8) | -0.18(-0.26--0.1) | **<.001** | 0.06(0.05-0.06) | -0.15(-0.27--0.03) | **.016** |
| **Model 3** |  |  |  |  |  |  |  |  |  |
| EoP |  |  |  |  |  |  |  |  |  |
| No-EoP |  | -0.09(-0.16--0.02) | **.012** |  | 0.01(-0.06-0.08) | 0.844 |  | -0.1(-0.2-0.01) | .063 |
|  |  |  |  |  |  |  |  |  |  |
| Vape |  |  |  |  |  |  |  |  |  |
| Smoke |  | 0.28(0.2-0.36) | **<.001** |  | 0.19(0.12-0.27) | **<.001** |  | 0.16(0.04-0.27) | **.008** |
| Dual |  | 0.25(0.17-0.34) | **<.001** |  | 0.16(0.07-0.25) | **<.001** |  | 0.08(-0.05-0.21) | .238 |
| Non-use |  | -0.26(-0.34--0.18) | **<.001** |  | -0.17(-0.25--0.09) | **<.001** |  | -0.13(-0.25--0.01) | **.037** |
|  |  |  |  |  |  |  |  |  |  |
| Cannabis use | 2.1(2-2.2) |  |  | 3.5(3.4-3.7) |  |  | 0.07(0.07-0.08) |  |  |
| No-cannabis use | 2.4(2.3-2.5) | -0.01(-0.05-0.03) | .590 | 3.4(3.4-3.5) | -0.07(-0.11--0.03) | **.002** | 0.07(0.06-0.07) | -0.09(-0.15--0.03) | **.003** |
| Biomarker levels were adjusted for mg/dL creatinine.  All analyses were adjusted for age, sex and ethnicity.  *analysis was missing for some participant samples.  BoEs are presented by group and then ordered alphabetically. | | | | | | | | | |

| Table S5: associations between biomarker levels, EoP, Vaping/smoking and cannabis pg/ml creatinine (N=5750) | | | | | | | | | |
| --- | --- | --- | --- | --- | --- | --- | --- | --- | --- |
|  | 34MH ng/ml (n=5750) | | | 3-HPMA ng/ml (n=5750) | | | AAMA ng/ml (n=5750) | | |
|  | GM(95% CI) | Beta (95% CI) | p | GM(95% CI) | Beta (95% CI) | p | GM(95% CI) | Beta (95% CI) | p |
| Total | 3231.5(3134.8-3331.3) |  |  | 7368(7154.6-7587.6) |  |  | 996.8(976-1018) |  |  |
| **Model 1** |  |  |  |  |  |  |  |  |  |
| EoP | 4343.9(3870.2-4875.5) |  |  | 9910.7(8794-11169.1) |  |  | 1195.2(1097.4-1301.7) |  |  |
| No-EoP | 3168.1(3070.1-3269.3) | -0.35(-0.47--0.22) | **<.001** | 7223.1(7007.9-7444.9) | -0.34(-0.46--0.22) | **<.001** | 984.7(963.6-1006.3) | -0.19(-0.28--0.11) | **<.001** |
| **Model 2** |  |  |  |  |  |  |  |  |  |
| EoP |  |  |  |  |  |  |  |  |  |
| No-EoP |  | -0.06(-0.15-0.04) | .262 |  | -0.05(-0.15-0.04) | .274 |  | -0.02(-0.09-0.06) | .676 |
|  |  |  |  |  |  |  |  |  |  |
| Vape | 1480.1(1333.2-1643.2) |  |  | 3551.5(3257.3-3872.3) |  |  | 609.1(557.9-665.1) |  |  |
| Smoke | 5991.6(5789.5-6200.8) | 1.34(1.23-1.45) | **<.001** | 13756.6(13275.7-14254.8) | 1.29(1.19-1.4) | **<.001** | 1434(1399.4-1469.5) | 0.85(0.77-0.94) | **<.001** |
| Dual | 4451.5(4132.5-4795.2) | 1.11(0.99-1.23) | **<.001** | 9646.6(8997.7-10342.2) | 1(0.89-1.12) | **<.001** | 1271.6(1203.1-1344) | 0.73(0.64-0.82) | **<.001** |
| Non-use | 1272.9(1222.7-1325.1) | -0.18(-0.29--0.07) | **.001** | 2909.4(2815.3-3006.7) | -0.24(-0.34--0.13) | **<.001** | 565.8(548.8-583.2) | -0.08(-0.16-0) | .063 |
| **Model 3** |  |  |  |  |  |  |  |  |  |
| EoP |  |  |  |  |  |  |  |  |  |
| No-EoP |  | -0.04(-0.14-0.06) | .424 |  | -0.05(-0.14-0.05) | .331 |  | 0.02(-0.05-0.09) | .646 |
|  |  |  |  |  |  |  |  |  |  |
| Vape |  |  |  |  |  |  |  |  |  |
| Smoke |  | 1.34(1.23-1.45) | **<.001** |  | 1.29(1.19-1.4) | **<.001** |  | 0.86(0.78-0.94) | **<.001** |
| Dual |  | 1.09(0.97-1.21) | **<.001** |  | 1(0.88-1.11) | **<.001** |  | 0.68(0.59-0.77) | **<.001** |
| Non-use |  | -0.14(-0.25--0.03) | **.014** |  | -0.22(-0.33--0.11) | **<.001** |  | 0(-0.08-0.09) | .938 |
|  |  |  |  |  |  |  |  |  |  |
| Cannabis use | 4447.1(4214.9-4692.2) |  |  | 9316.7(8816.2-9845.7) |  |  | 1525.7(1464.3-1589.6) |  |  |
| No-cannabis use | 2900(2797.8-3005.8) | -0.21(-0.27--0.15) | **<.001** | 6804.4(6575.1-7041.7) | -0.07(-0.13--0.02) | **.008** | 862.8(843.2-882.9) | -0.42(-0.46--0.38) | **<.001** |
| Biomarker levels were adjusted for mg/dL creatinine.  All analyses were adjusted for age, sex and ethnicity.  BoEs are presented by group and then ordered alphabetically. | | | | | | | | | |

| Table S6: associations between biomarker levels, EoP, Vaping/smoking and cannabis pg/ml creatinine (N=5750) | | | | | | | | | |
| --- | --- | --- | --- | --- | --- | --- | --- | --- | --- |
|  | AMCA ng/ml (n=5750) | | | CEMA ng/ml (n=5750) | | | CYMA ng/ml (n=5750) | | |
|  | GM(95% CI) | Beta (95% CI) | p | GM(95% CI) | Beta (95% CI) | p | GM(95% CI) | Beta (95% CI) | p |
| Total | 2635.8(2568.4-2705.1) |  |  | 1730.1(1692.5-1768.6) |  |  | 235(220.7-250.3) |  |  |
| **Model 1** |  |  |  |  |  |  |  |  |  |
| EoP | 3524.7(3202.9-3878.9) |  |  | 1980.2(1813.8-2161.9) |  |  | 555.8(444.2-695.5) |  |  |
| No-EoP | 2585(2516.6-2655.3) | 0.04(-0.04-0.11) | .347 | 1714.6(1676-1754) | 0.04(-0.04-0.11) | .347 | 221.9(207.8-236.8) | -0.93(-1.19--0.67) | **<.001** |
| **Model 2** |  |  |  |  |  |  |  |  |  |
| EoP |  |  |  |  |  |  |  |  |  |
| No-EoP |  | 0.04(-0.04-0.12) | .311 |  | 0.04(-0.04-0.12) | .311 |  | -0.13(-0.29-0.03) | .107 |
|  |  |  |  |  |  |  |  |  |  |
| Vape | 1449.2(1340.4-1566.9) |  |  | 1049(973.4-1130.4) |  |  | 33.1(27.7-39.7) |  |  |
| Smoke | 4532.3(4404.1-4664.3) | 0.05(-0.04-0.13) | .293 | 2592.9(2521.6-2666.2) | 0.05(-0.04-0.13) | .293 | 1192.8(1131.7-1257.1) | 3.51(3.33-3.68) | **<.001** |
| Dual | 3481.7(3272.5-3704.2) | 0.01(-0.09-0.11) | .851 | 2019.7(1910.4-2135.2) | 0.01(-0.09-0.11) | .851 | 678.3(597.7-769.9) | 3.01(2.81-3.21) | **<.001** |
| Non-use | 1148.7(1109.8-1188.9) | 0.02(-0.07-0.1) | .733 | 959.5(932.1-987.8) | 0.02(-0.07-0.1) | .733 | 18.2(17.1-19.4) | -0.65(-0.83--0.47) | **<.001** |
| **Model 3** |  |  |  |  |  |  |  |  |  |
| EoP |  |  |  |  |  |  |  |  |  |
| No-EoP |  | 0.04(-0.04-0.12) | .323 |  | 0.04(-0.04-0.12) | .323 |  | -0.07(-0.23-0.08) | .371 |
|  |  |  |  |  |  |  |  |  |  |
| Vape |  |  |  |  |  |  |  |  |  |
| Smoke |  | 0.05(-0.04-0.13) | .294 |  | 0.05(-0.04-0.13) | .294 |  | 3.51(3.34-3.69) | **<.001** |
| Dual |  | 0.01(-0.09-0.11) | .828 |  | 0.01(-0.09-0.11) | .828 |  | 2.92(2.73-3.11) | **<.001** |
| Non-use |  | 0.01(-0.08-0.1) | .777 |  | 0.01(-0.08-0.1) | .777 |  | -0.49(-0.67--0.32) | **<.001** |
|  |  |  |  |  |  |  |  |  |  |
| Cannabis use | 3208.6(3066.6-3357.2) |  |  | 1919.3(1842-1999.8) |  |  | 752.2(685.9-824.9) |  |  |
| No-cannabis use | 2465.8(2390.8-2543.2) | 0.01(-0.03-0.06) | .576 | 1670.3(1627.6-1714.1) | 0.01(-0.03-0.06) | .576 | 158.4(147-170.8) | -0.77(-0.86--0.68) | **<.001** |
| Biomarker levels were adjusted for mg/dL creatinine.  All analyses were adjusted for age, sex and ethnicity.  BoEs are presented by group and then ordered alphabetically. | | | | | | | | | |

| Table S7: associations between biomarker levels, EoP, Vaping/smoking and cannabis pg/ml creatinine (N=5750) | | | | | | | | | |
| --- | --- | --- | --- | --- | --- | --- | --- | --- | --- |
|  | HPM2 ng/ml (n=5750) | | | HEMA ng/ml (n=5750) | | | HPMM ng/ml (n=5750) | | |
|  | GM(95% CI) | Beta (95% CI) | p | GM(95% CI) | Beta (95% CI) | p | GM(95% CI) | Beta (95% CI) | p |
| Total | 546.4(534-559.1) |  |  | 19.9(19.4-20.5) |  |  | 6247.6(6067.5-6432.9) |  |  |
| **Model 1** |  |  |  |  |  |  |  |  |  |
| EoP | 652.4(594.6-715.9) |  |  | 25(22.4-28.1) |  |  | 8678.6(7733.6-9739.1) |  |  |
| No-EoP | 540(527.3-552.9) | -0.2(-0.3--0.11) | **<.001** | 19.6(19-20.2) | -0.23(-0.34--0.12) | **<.001** | 6111.5(5930-6298.6) | -0.38(-0.49--0.26) | **<.001** |
| **Model 2** |  |  |  |  |  |  |  |  |  |
| EoP |  |  |  |  |  |  |  |  |  |
| No-EoP |  | -0.05(-0.14-0.03) | .218 |  | -0.03(-0.13-0.07) | .498 |  | -0.09(-0.18-0) | **.048** |
|  |  |  |  |  |  |  |  |  |  |
| Vape | 361(330.5-394.2) |  |  | 11.1(10.1-12.2) |  |  | 2344.2(2196.8-2501.3) |  |  |
| Smoke | 758.6(737.1-780.6) | 0.72(0.62-0.81) | **<.001** | 30.4(29.2-31.5) | 0.99(0.88-1.1) | **<.001** | 12019.5(11603.1-12450.9) | 1.55(1.45-1.65) | **<.001** |
| Dual | 633.2(597-671.6) | 0.56(0.46-0.67) | **<.001** | 24.5(22.8-26.3) | 0.77(0.65-0.89) | **<.001** | 8093.9(7529-8701.1) | 1.25(1.14-1.35) | **<.001** |
| Non-use | 336.2(323.6-349.3) | -0.09(-0.19-0.01) | .080 | 10.7(10.3-11) | -0.08(-0.19-0.04) | .177 | 2465.2(2395.8-2536.6) | 0(-0.1-0.1) | 0.954 |
| **Model 3** |  |  |  |  |  |  |  |  |  |
| EoP |  |  |  |  |  |  |  |  |  |
| No-EoP |  | -0.05(-0.13-0.04) | .258 |  | -0.03(-0.13-0.07) | .576 |  | -0.08(-0.17-0.01) | .071 |
|  |  |  |  |  |  |  |  |  |  |
| Vape |  |  |  |  |  |  |  |  |  |
| Smoke |  | 0.72(0.62-0.82) | **<.001** |  | 0.99(0.88-1.1) | **<.001** |  | 1.55(1.45-1.65) | **<.001** |
| Dual |  | 0.56(0.45-0.66) | **<.001** |  | 0.76(0.64-0.89) | **<.001** |  | 1.23(1.12-1.34) | **<.001** |
| Non-use |  | -0.08(-0.17-0.02) | .128 |  | -0.06(-0.18-0.05) | .281 |  | 0.02(-0.08-0.12) | 0.747 |
|  |  |  |  |  |  |  |  |  |  |
| Cannabis use | 619.7(594.1-646.5) |  |  | 24(22.8-25.3) |  |  | 7818.9(7404.6-8256.4) |  |  |
| No-cannabis use | 523.6(509.6-538) | -0.06(-0.11--0.01) | **.026** | 18.7(18.1-19.3) | -0.08(-0.14--0.02) | **.009** | 5789.9(5595-5991.6) | -0.1(-0.15--0.05) | **<.001** |
| Biomarker levels were adjusted for mg/dL creatinine.  All analyses were adjusted for age, sex and ethnicity.  BoEs are presented by group and then ordered alphabetically. | | | | | | | | | |

| Table S8: associations between biomarker levels, EoP, Vaping/smoking and cannabis pg/ml creatinine (N=5750) | | | | | | | | | |
| --- | --- | --- | --- | --- | --- | --- | --- | --- | --- |
|  | IPM3 ng/ml (n=5750) | | | MHB3 ng/ml (n=5750) | | | MADA ng/ml (n=5750) | | |
|  | GM(95% CI) | Beta (95% CI) | p | GM(95% CI) | Beta (95% CI) | p | GM(95% CI) | Beta (95% CI) | p |
|  |  |  |  |  |  |  |  |  |  |
| Total | 145.7(140.2-151.4) |  |  | 154.8(150-159.8) |  |  | 2359.4(2318.7-2401) |  |  |
| **Model 1** |  |  |  |  |  |  |  |  |  |
| EoP | 236.3(204-273.6) |  |  | 222.8(197.2-251.6) |  |  | 2859.4(2656.2-3078) |  |  |
| No-EoP | 141(135.5-146.7) | -0.54(-0.7--0.39) | **<.001** | 151.1(146.2-156.1) | -0.41(-0.54--0.28) | **<.001** | 2329.3(2288-2371.3) | -0.22(-0.29--0.15) | **<.001** |
| **Model 2** |  |  |  |  |  |  |  |  |  |
| EoP |  |  |  |  |  |  |  |  |  |
| No-EoP |  | -0.14(-0.25--0.02) | **.019** |  | -0.07(-0.16-0.02) | .112 |  | -0.09(-0.15--0.03) | **.005** |
|  |  |  |  |  |  |  |  |  |  |
| Vape | 42.1(38.5-46) |  |  | 50.2(46.6-54) |  |  | 1663.7(1551.9-1783.6) |  |  |
| Smoke | 355.2(339.9-371.1) | 2.05(1.92-2.18) | **<.001** | 327.2(315.9-339) | 1.81(1.71-1.91) | **<.001** | 3158.7(3087.7-3231.4) | 0.6(0.53-0.67) | **<.001** |
| Dual | 220(200.1-241.9) | 1.66(1.52-1.8) | **<.001** | 216(199.8-233.6) | 1.46(1.35-1.58) | **<.001** | 2608.7(2495.5-2727) | 0.45(0.37-0.53) | **<.001** |
| Non-use | 39.5(38-40.9) | -0.12(-0.25-0.01) | 0.073 | 52.6(51.1-54.2) | 0(-0.1-0.11) | .974 | 1546.7(1510-1584.3) | -0.1(-0.17--0.03) | **.004** |
| **Model 3** |  |  |  |  |  |  |  |  |  |
| EoP |  |  |  |  |  |  |  |  |  |
| No-EoP |  | -0.13(-0.24--0.01) | **.027** |  | -0.07(-0.16-0.02) | .143 |  | -0.08(-0.14--0.02) | **.010** |
|  |  |  |  |  |  |  |  |  |  |
| Vape |  |  |  |  |  |  |  |  |  |
| Smoke |  | 2.05(1.92-2.18) | **<.001** |  | 1.81(1.71-1.91) | **<.001** |  | 0.6(0.53-0.67) | **<.001** |
| Dual |  | 1.64(1.5-1.79) | **<.001** |  | 1.45(1.34-1.57) | **<.001** |  | 0.44(0.36-0.52) | **<.001** |
| Non-use |  | -0.1(-0.23-0.03) | 0.138 |  | 0.02(-0.09-0.12) | .754 |  | -0.09(-0.16--0.02) | **.015** |
|  |  |  |  |  |  |  |  |  |  |
| Cannabis use | 201.9(188.1-216.7) |  |  | 200.8(189.7-212.6) |  |  | 2648.2(2564-2735.1) |  |  |
| No-cannabis use | 130.4(124.6-136.4) | -0.1(-0.17--0.03) | **.003** | 141.7(136.5-147.1) | -0.08(-0.13--0.02) | **.005** | 2268.9(2222.8-2315.9) | -0.08(-0.11--0.04) | **<.001** |
| Biomarker levels were adjusted for mg/dL creatinine.  All analyses were adjusted for age, sex and ethnicity.  BoEs are presented by group and then ordered alphabetically. | | | | | | | | | |

| Table S9: associations between biomarker levels, EoP, Vaping/smoking and cannabis pg/ml creatinine (N=5750) | | | | | | |
| --- | --- | --- | --- | --- | --- | --- |
|  | PHGA ng/ml (n=5750) | | | PMA ng/ml (n=5750) | | |
|  | GM(95% CI) | Beta (95% CI) | p | GM(95% CI) | Beta (95% CI) | p |
|  |  |  |  |  |  |  |
| Total | 2964(2915.7-3013.1) |  |  | 8.8(8.6-8.9) |  |  |
| **Model 1** |  |  |  |  |  |  |
| EoP | 3379.4(3149.2-3626.4) |  |  | 8.3(7.7-9) |  |  |
| No-EoP | 2938.1(2889-2988.1) | -0.16(-0.22--0.09) | **<.001** | 8.8(8.6-9) | 0.04(-0.04-0.11) | .347 |
| **Model 2** |  |  |  |  |  |  |
| EoP |  |  |  |  |  |  |
| No-EoP |  | -0.05(-0.11-0.01) | .124 |  | 0.04(-0.04-0.12) | .311 |
|  |  |  |  |  |  |  |
| Vape | 2232(2083.3-2391.2) |  |  | 8.4(7.7-9.2) |  |  |
| Smoke | 3752.7(3671.4-3835.7) | 0.49(0.42-0.56) | **<.001** | 9(8.7-9.2) | 0.05(-0.04-0.13) | .293 |
| Dual | 3322(3179.9-3470.4) | 0.4(0.33-0.48) | **<.001** | 8.4(8-8.9) | 0.01(-0.09-0.11) | .851 |
| Non-use | 2078.5(2029.9-2128.4) | -0.09(-0.16--0.02) | **.009** | 8.7(8.4-9) | 0.02(-0.07-0.1) | .733 |
| **Model 3** |  |  |  |  |  |  |
| EoP |  |  |  |  |  |  |
| No-EoP |  | -0.04(-0.1-0.02) | .211 |  | 0.04(-0.04-0.12) | .323 |
|  |  |  |  |  |  |  |
| Vape |  |  |  |  |  |  |
| Smoke |  | 0.49(0.42-0.56) | **<.001** |  | 0.05(-0.04-0.13) | .294 |
| Dual |  | 0.39(0.32-0.46) | **<.001** |  | 0.01(-0.09-0.11) | .828 |
| Non-use |  | -0.07(-0.14-0) | .053 |  | 0.01(-0.08-0.1) | .777 |
|  |  |  |  |  |  |  |
| Cannabis use | 3363.8(3265.4-3465.2) |  |  | 8.3(8-8.6) |  |  |
| No-cannabis use | 2839.5(2785-2895.2) | -0.11(-0.15--0.08) | **<.001** | 8.9(8.7-9.1) | 0.01(-0.03-0.06) | .576 |
| Biomarker levels were adjusted for mg/dL creatinine.  All analyses were adjusted for age, sex and ethnicity.  BoEs are presented by group and then ordered alphabetically. | | | | | | |

| Table S10: Geometric Mean levels of biomarkers among participants with and without EoP | | | | | | | | |
| --- | --- | --- | --- | --- | --- | --- | --- | --- |
|  | EoP | | | | No-EoP | | | |
|  | Vaped (n=18) | Smoked (n=215) | Dual  (n=77) | No-use  (n=51) | Vaped (n=279) | Smoked (n=2588) | Dual (n=698) | No-use (n=1824) |
| Nicotine ng/ml | | | | | | | | |
| Cotinine | 16.18 | 21.62 | 12.00 | **0.01** | 4.03 | **17.21** | **13.99** | **.005** |
| 3-HC | 29.36 | 37.70 | 22.40 | **0.01** | 6.98 | **30.05** | **22.67** | **0.01** |
| TSNA ng/ml | | | | | | | | |
| NNAL | .00005 | **.00263** | **.00102** | **.00001** | .00003 | **.00221** | **.00105** | **.00001** |
| NNN | .00003 | **.00007** | **.00006** | .00002 | .00003 | **.00007** | **.00006** | **.00002** |
| Metals ug/L | | | | | | | | |
| Lead | .0033 | .0040 | .0036 | .0025 | .0030 | **.0041** | **.0035** | **.0027** |
| Cadmium | .0020 | **.0031** | **.0027** | .0016 | .0017 | **.0030** | **.0022** | **.0016** |
| Uranium | .00007 | .00008 | .00008 | .00006 | .00006 | **.00008** | .00007 | **.00005** |
| VOCs ng/ml | | | | | | | | |
| 3-HPMA | 4.06 | **14.81** | **9.53** | 2.65 | 3.52 | **13.67** | **9.66** | **2.92** |
| 34MH | 2.42 | **6.01** | **4.67** | **1.22** | 1.43 | **5.99** | **4.43** | 1.27 |
| AAMA | 0.42 | **1.53** | **1.32** | 0.52 | 0.62 | **1.43** | **1.27** | 0.57 |
| AMCA | 1.48 | 4.77 | 3.87 | 1.16 | 1.45 | 4.51 | 3.44 | 1.15 |
| CEMA | 0.94 | 2.56 | 2.03 | 0.83 | 1.06 | 2.6 | 2.02 | 0.96 |
| CYMA | 0.04 | **1.43** | **0.64** | 0.02 | 0.03 | **1.17** | **0.68** | **0.02** |
| HEMA | 0.01 | **0.03** | **0.03** | 0.01 | 0.01 | **0.03** | **0.02** | 0.01 |
| HPM2 | 0.25 | **0.82** | **0.69** | 0.31 | 0.37 | **0.75** | **0.63** | 0.34 |
| HPMM | 2.61 | **13.44** | **8.23** | 2.27 | 2.33 | **11.91** | **8.08** | 2.47 |
| IPM3 | 0.05 | **0.4** | **0.24** | 0.04 | 0.04 | **0.35** | **0.22** | 0.04 |
| MADA | 1.66 | **3.43** | **2.78** | 1.67 | 1.66 | **3.14** | **2.59** | **1.54** |
| MHB3 | 0.05 | **0.36** | **0.23** | 0.05 | 0.05 | **0.32** | **0.21** | 0.05 |
| PHGA | 2.28 | **3.87** | **3.38** | 2.2 | 2.23 | **3.74** | **3.32** | 2.08 |
| PMA | .0076 | .0085 | .0087 | .0070 | .0085 | .0090 | .0084 | .0087 |
| Biomarker levels were adjusted for mg/dL creatinine.  **Bold** denotes significantly different to vaping by p<.05 from model 3 interaction  Linear regression analysis adjusted for age, sex, ethnicity and past 30-day cannabis use  All toxicant levels were adjusted for creatinine | | | | | | | | |

| Table S11: associations between biomarker levels, EoP, and vaping characteristics (Vaping only N=297) | | | | | | |
| --- | --- | --- | --- | --- | --- | --- |
|  | Cotinine | | | 3_HC | | |
|  | GM  (95% CI) | Beta  (95% CI) | p | GM  (95% CI) | Beta  (95% CI) | p |
| **Psychosis** |  |  |  |  |  |  |
| EoP |  | 0 | REF |  | 0 | REF |
| No-EoP |  | -1.61(-3.26- 0.05) | .057 |  | -1.59(-3.25-0.07) | .062 |
| **Device type (279)b** | |  |  |  |  |  |
| Tank | 6.31(4.08-9.78) | 0 | REF | 10.88(7.07-16.73) | 0 | REF |
| Pod | 3.73(1.83-7.63) | -0.42(-1.24- 0.41) | .324 | 6.45(3.01-13.84) | -0.43(-1.27-0.40) | .309 |
| Disposable | 0.25(0.001-9.08) | -3.65(-5.90- -1.42) | **.030** | 0.43(0.02-13.96) | -3.72(-5.98- -1.46) | **.001** |
|  |  |  |  |  |  |  |
| **Psychosis** |  |  |  |  |  |  |
| EoP |  | 0 | Ref |  | 0 | Ref |
| No-EoP |  | -0.54(-1.71- 0.64) | .368 |  | -0.54(-1.7- 0.65) | .372 |
| **Nicotine concentration (N=242)c** | |  |  |  |  |  |
| 13mg+ | 22.09(13.17-37.06) | 0 | Ref | 34.58(19.66-60.83) | 0 | Ref |
| 1-12mg | 15.74(11.64-21.28) | -0.42(-1.07-0.24) | .214 | 27.25(20.08-36.97) | -0.33(-0.99-0.34) | .337 |
| 0mg | 0.04(0.02-0.11) | -6.22(-7.12—5.31) | **<.001** | 0.08(0.03-0.21) | 06.10(-7.01- 05.17) | **<.001** |
| Biomarker levels were adjusted for mg/dL creatinine.  Models are adjusted for age, sex, ethnicity and past 30-day cannabis use  aPeople using NRT in the past 12 months were removed  bParticipants who did not know their device type were removed  cParticipants who reported don’t know or refused to nicotine concentration vaped question were remove | | | | | | |

| Table S12: associations between biomarker levels, EoP, and vaping characteristics (Vaping only N=297) | | | | | | | | | |
| --- | --- | --- | --- | --- | --- | --- | --- | --- | --- |
|  | AAMA | | | _34MH | | | HPM2 | | |
|  | GM  (95% CI) | Beta  (95% CI) | p | GM  (95% CI) | Beta  (95% CI) | p | GM  (95% CI) | Beta  (95% CI) | p |
| **Psychosis** |  |  |  |  |  |  |  |  |  |
| EoP |  | 0 | Ref |  | 0 | Ref |  | 0 | Ref |
| No-EoP |  | 0.42  (0.05-0.77) | **.025** |  | -0.48  (-0.91- -0.05) | **.030** |  | 0.44  (0.07-0.81) | **.020** |
| **Device type (n=295)a** | |  |  |  |  |  |  |  |  |
| Tank | 0.59  (0.53-0.65) | 0 | Ref | 2.00  (1.06-3.77) | 0 | Ref | 0.37  (0.26-0.52) | 0 | Ref |
| Pod | 0.62  (0.54-0.73) | 0.001  (-0.19- 0.19) | .988 | 1.41  (1.18-1.69) | -0.07(-0.30-0.15) | .523 | 0.33(  0.28-0.38) | -0.17  (-0.36-0.03) | .092 |
| Disposable | 1.15  (0.54-2.46) | 0.59  (0.09- 1.10) | **.021** | 1.51  (1.32-1.72) | 0.27(-0.33-0.87) | .377 | 0.38  (0.34-0.43) | -0.06  (-0.57- 0.46) | .813 |
|  |  |  |  |  |  |  |  |  |  |
| **Psychosis** |  |  |  |  |  |  |  |  |  |
| EoP |  | 0 | Ref |  | 0 | Ref |  | 0 | Ref |
| No-EoP |  | 0.45  (0.07-0.83) | **.012** |  | -0.54  (-0.98- -0.09) | **.018** |  | 0.39  (0.01-0.77) | **.042** |
| **Nicotine concentration (n=250)b** | | |  |  |  |  |  |  |  |
| 13mg+ | 0.66  (0.54-0.78) | 0 | Ref | 1.32  (1.04-1.67) | 0 | Ref | 0.36  (0.30-0.43) | 0 | Ref |
| 1-12mg | 0.59  (0.52-0.67) | -0.05  (-0.27-0.17) | .667 | 1.62  (1.39-1.88) | 0.26  (-0.003- 0.52) | .053 | 0.38  (0.33-0.43) | 0.04  (-0.18- 0.27) | .685 |
| 0mg | 0.53  (0.42-0.67) | -0.28  (-0.59-0.03) | .078 | 1.23  (0.98-1.54) | -0.07  (-0.43- 0.30) | .722 | 0.30  (0.25-0.38) | -0.21  (-0.52- 0.09) | .176 |
| Biomarker levels were adjusted for mg/dL creatinine.  Models are adjusted for age, sex, ethnicity and past 30-day cannabis use  aParticipants who did not know their device type were removed  bParticipants who reported don’t know or refused to nicotine concentration vaped question were remove | | | | | | | | | |

| Table S13: associations between biomarker levels, EoP, and heaviness of smoking (Smoking only N=2165) | | | | | | | | | | | | | |
| --- | --- | --- | --- | --- | --- | --- | --- | --- | --- | --- | --- | --- | --- |
|  | Cadmium | | | HPMM | | | IPM3 | | | MADA | | | |
|  | GM  (95% CI) | Beta  (95% CI) | p | GM  (95% CI) | Beta  (95% CI) | p | GM(95% CI) | Beta (95% CI) | p | GM(95% CI) | Beta  (95% CI) | p |  |
| **Psychosis** |  |  |  |  |  |  |  |  |  |  |  |  |  |
| EoP | 0.003  (0.003-0.004) | 0 | ref | 13.44  (11.93-15.14) | 0 | ref | 0.40  (0.34-0.47) | 0 | ref | 3.43  (3.15-3.74) | 0 | ref |  |
| No-EoP | 0.003  (0.003-0.003) | -0.03(-0.13-0.06) | .485 | 11.90  (11.48-12.35) | 0.50(-0.07- 0.14) | .499 | 0.35  (0.34-0.37) | 0.67(-0.31-0.20) | .665 | 3.14  (3.06-3.21) | -0.04(-0.13- 0.04) | .299 |  |
| **HSI** |  |  |  |  |  |  |  |  |  |  |  |  |  |
| Low | 0.002  (0.002-0.003) | 0 | ref | 9.74  (9.0-10.48) | 0 | ref | 0.28  (0.26-0.31) | 0 | ref | 2.85  (2.70-3.00) | 0 | ref |  |
| Moderate | 0.003  (0.003-0.004) | 0.25(0.18-0.31) | **<.001** | 17.40  (16.83-17.99) | 0.53(0.46-0.60) | **<.001** | 0.57  (0.55-0.59) | 0.58(0.38-0.77) | **<.001** | 3.65  (3.55-3.75) | 0.21(0.17-0.27) | **<.001** |  |
| High | 0.005  (0.005-0.006) | 0.50(0.39-0.61) | **<.001** | 25.21  (23.3-27.3) | 0.84(0.73-0.96) | **<.001** | 0.78  (0.71-0.86)) | 1.31(0.93-1.70) | **<.001** | 4.67  (4.33-5.04) | 0.42(0.32-0.52) | **<.001** |  |
| Models are adjusted for age, sex, ethnicity and past 30-day cannabis use | | | | | | | | | | | | | |
