# Supplementary material for: Tobacco-related toxicant exposure among people with and without experience of psychosis: findings from the US Population Assessment of Tobacco and Health study
Source: BMJ Open. 2025 Oct 10;15(10):e101066. doi: 10.1136/bmjopen-2025-101066 (PMC12517016; doi:10.1136/bmjopen-2025-101066)
Supplement: Supplementary file 2 [file bmjopen-2025-101066.reviewer_comments.pdf]

PEER REVIEW HISTORY

BMJ Open publishes all reviews undertaken for accepted manuscripts. Reviewers are asked to complete a checklist review form and are provided with free text boxes to elaborate on their assessment. These free text comments are reproduced below.

ARTICLE DETAILS

Title (Provisional)

Tobacco-related toxicant exposure among people with and without experience of psychosis; findings from the US Population Assessment of Tobacco and Health study

Authors

Taylor, Eve; Marczylo, Tim; East, Katherine; Robson, Deborah; McNeill, Ann; Tattan-Birch, Harry

---

VERSION 1 - REVIEW

Reviewer 1

Name Ward, Heather

Affiliation Vanderbilt University Medical Center, Psychiatry and Behavioral Sciences

Date 10-May-2025

COI None

Individuals with psychosis have a significantly higher prevalence of tobacco use compared to the general population, which leads to a 20-year decreased life expectancy. It is well-documented that people with psychosis have greater levels of nicotine dependence and higher levels of nicotine metabolites. However, it is unknown if levels of toxicants from nicotine use are also significantly higher. In this manuscript, authors performed a secondary analysis of a large, publicly available dataset to quantify levels of nicotine metabolites and toxicants among people who smoked or vaped based on self-reported history of psychosis. Indeed, authors find that people with a history of psychosis have significantly higher levels of nicotine toxicants. However, this effect was no longer significant after controlling for nicotine use, thereby demonstrating that higher toxicant levels are attributable to the higher levels of nicotine use by people with psychosis. This is a well-designed analysis and well-written manuscript. I only have a few minor suggestions. Although the purpose of this manuscript was to examine the effect of experience of psychosis, I also think it would be helpful to comment more on the toxicant differences between mode of nicotine use. Vaping did not appear to have significantly higher levels of toxicants compared to nonuse. Although the assessment of cannabis use did not specify mode of use (i.e., smoking vs edibles; flower vs

extract), it may also be interesting to see if cannabis and nicotine co-use significantly increases toxicant levels.

**Reviewer**	**2**
**Name**	**Levey, Daniel**
**Affiliation**	**Yale University SASC**
**Date**	**27-May-2025**
**COI**	**None**

An investigation of nicotine-related toxicant levels and experience of psychosis in the PATH cohort. This relationship is of public health concern.

Experience of psychosis "told by health professional that you have schizophrenia, SZA, psychosis, psychotic illness, or psychotic episode." 6.3% of participants (361) had this status. Power? Why include 20 individuals of uncertain status (Don't know/refused to answer) as controls? There is a huge 16:1 ratio of controls to cases.

Similar issue, given the large difference in ratio between EoP and no-EoP no-use (14% and 34% of those groups, respectively), might it be worth downsampling no-EoP individuals to better match reported nicotine (or cannabis) use?

For justifying the use of geometric means, could the distributions be provided in the supplement? At least for primary findings?

Biomarkers are not well-defined here. They are biomarkers for what? Probably there should be some background or context provided for how and why these were used? What is the known relationship of those biomarkers/toxicants to nicotine/cannabis/EoP? The term biomarker seems to be used interchangeably with toxicants, is this intended?

Deviation from pre-registration in adjusting for cannabis use in follow-up models. If cannabis use attenuated the relationship with vaping or smoking, so observed differences could be mediated by cannabis. This is in discussion but could be mentioned earlier. Any replication samples from UK or Europe, if nicotine/cannabis comorbidity is indeed lower? Is an implication that increased cadmium and uranium among EoP participants is mediated by cannabis, given the results comparing model 2 and model 3?

VERSION 1 - AUTHOR RESPONSE

Dr. Heather Ward, Vanderbilt University Medical Center

Comments to the Author:

Individuals with psychosis have a significantly higher prevalence of tobacco use compared to the general population, which leads to a 20-year decreased life expectancy. It is well-documented that people with psychosis have greater levels of nicotine dependence and higher levels of nicotine metabolites. However, it is unknown if levels of toxicants from nicotine use are also significantly higher. In this manuscript, authors performed a secondary analysis of a large, publicly available dataset to quantify levels of nicotine metabolites and toxicants among people who smoked or vaped based on self-reported history of psychosis. Indeed, authors find that people with a history of psychosis have significantly higher levels of nicotine toxicants. However, this effect was no longer significant after controlling for nicotine use, thereby demonstrating that higher toxicant levels are attributable to the higher levels of nicotine use by people with psychosis. This is a well-designed analysis and well-written manuscript. I only have a few minor suggestions.

Thank you very much for your review

Although the purpose of this manuscript was to examine the effect of experience of psychosis, I also think it would be helpful to comment more on the toxicant differences between mode of nicotine use. Vaping did not appear to have significantly higher levels of toxicants compared to nonuse.

Thank you for this suggestion, we have added a comment on this to the discussion.

Summary paragraph:

“Across all participants, levels of TSNAs, metals and most VOCIs were significantly lower among those who vaped compared to those smoked or dual used.”

Additional discussion:

“Across all participants, levels of TSNAs, metals and most VOCs were significantly lower among people who vaped compared to those who smoked or dual used, which is consistent with previous biomarker research (20,21). Levels of metals and some VOCs were greater among people who vaped compared to those who neither vaped nor smoked; however, so were levels of TSNAs and the VOC CYMA, both of which indicate recent tobacco exposure among people who had vaped (36). This supports the harm reduction potential of the use of e-cigarette as a smoking cessation tool for people with EoP, particularly as we found that they are exposed to significantly higher levels of cadmium and some VOCs due to their heaviness of tobacco smoking than people who smoke without EoP. Greater rates of cannabis consumption also contributed to higher levels of toxicant exposure. These findings emphasise the need for targeted stop-smoking support and cannabis harm reduction interventions for this community to reduce toxicant exposure and subsequent disease.”

Although the assessment of cannabis use did not specify mode of use (i.e., smoking vs edibles; flower vs extract), it may also be interesting to see if cannabis and nicotine co-use significantly increases toxicant levels.

Thank you for this comment. Model 3 adjusts for vaping/smoking, and cannabis use and thus can be used to determine associations between cannabis use and toxicant level when adjusting for other vaping/smoking. We have added extra detail on this to the results and the discussion:

Results

"Associations between toxicant exposure and cannabis

In model 3, when adjusting for EoP and smoking and vaping status, levels of lead, uranium, 34MH, 3-HPMA, AAMA, CYMA, HPM2, HEMA, HPMM, IMP3, MHB3, MADA and PHGA were significantly greater among people who used cannabis compared to people who did not (Tables S3-S9)."

Discussion

"Past 30-day cannabis use was associated with levels of lead, uranium, and most VOCs. These findings are consistent with previous research reporting high levels of VOCs in bio-samples of participants who smoke cannabis (16,17). Associations between EoP, uranium and the VOC HPMM lost significance when adjusting for cannabis use. This suggests that greater cannabis use among people with EoP contributes to higher exposure of these toxicants among this community. Cannabis use was not found to moderate levels of nicotine metabolites or TSNAs. This is consistent with findings of very low levels of NNAL and total nicotine equivalents among participants who exclusively smoke cannabis without tobacco (38). However, the present study used data from the US where mixing cannabis with tobacco is rare (39) and so findings may not generalise to other countries where mixing cannabis with tobacco is more common (e.g., England). "

Reviewer: 2

Dr. Daniel Levey, Yale University SASC

Comments to the Author:

An investigation of nicotine-related toxicant levels and experience of psychosis in the PATH cohort. This relationship is of public health concern.

Thank you very much for your review

Experience of psychosis "told by health professional that you have schizophrenia, SZA, psychosis, psychotic illness, or psychotic episode." 6.3% of participants (361) had this status. Power? Why include 20 individuals of uncertain status (Don't know/refused to answer) as controls? There is a huge 16:1 ratio of controls to cases. Similar issue, given the large difference in ratio between EoP and no-EoP no-use (14% and 34% of those groups, respectively), might it be worth down sampling no-EoP individuals to better match reported nicotine (or cannabis) use?

We have added to the limitations section that:

“The number of participants with a history of psychosis in the sample was relatively small (N=361), which may limit the precision of estimates for this group.”

However, we respectfully note that the concerns raised regarding power and the control-to-case ratio are not directly applicable; this is a cross-sectional analysis of data from the PATH study, which is designed to be representative of the US population, not a case control study. The observed 16:1 ratio of participants without versus with a history of psychosis reflects the low base rate of psychotic disorders in the general population, not an artefact of sampling. According to the National Institute of Mental Health, approximately 0.015–0.1% of the US population experiences psychosis annually. Downsampling would reduce representativeness of our sample. We have therefore chosen to maintain our samples (https://www.nimh.nih.gov/health/publications/understanding-psychosis).

We also included the 20 participants who responded “don’t know” or refused to answer in the comparison group, as they represent a very small proportion of this group (0.35%) and thus their inclusion or exclusion has a negligible impact on the results. Excluding them could introduce bias through unnecessary data loss. We have therefore retained them in the analysis.

For justifying the use of geometric means, could the distributions be provided in the supplement? At least for primary findings?

The use of geometric means is standard practice for summarising biomarker values, regardless of the degree of skew in the distribution. This is because biomarkers are strictly positive and often reflect underlying multiplicative biological processes. In such contexts, where variability or measurement error tends to scale with the magnitude of the value, geometric means offer a more stable and interpretable summary measure. They are particularly well suited for comparing ratios or relative differences across groups. This approach is widely endorsed in biomedical research, including in large-scale surveys such as NHANES and in methodological literature (e.g., Olivier et al., 2008), and remains appropriate even when distributions are not markedly skewed. We have therefore not added the distributions to the manuscript.

https://doi.org/10.1016/S1081-1206(10)60595-9

Biomarkers are not well-defined here. They are biomarkers for what? Probably there should be some background or context provided for how and why these were used? The term biomarker seems to be used interchangeably with toxicants, is this intended?

We have now amended this so the term ‘toxicant’ is used throughout apart from in the introduction where the terms are introduced. We have also added the following detail and definitions to the introduction

“Smoking-related diseases are caused by exposure to numerous carcinogenic, cardiovascular, and respiratory toxicants found in cigarettes and tobacco smoke. It is estimated that tobacco smoke contains 93 harmful and potentially harmful constituents (11), many of which can be classed as; Tobacco Specific Nitrosamines (TSNAs), Volatile Organic Compounds (VOCs), Polycyclic Aromatic

Hydrocarbons (PAHs), metals, and nicotine (see Table 1). Exposure to these toxicants can be estimated by measuring the levels of these toxicants, or their metabolites in the bio samples of people who smoke, commonly referred to as their biomarkers of exposure. The term toxicant will be used throughout to refer to toxicants and their biomarkers of exposure."

What is the known relationship of those biomarkers/toxicants to nicotine/cannabis/EoP?

We have added details on this to the introduction

"Cannabis use, with and without tobacco, can also expose people to these harmful toxicants. High levels of VOCs have been reported in the bio-samples of participants who smoke cannabis (12,13) and are also reported in the aerosol of vaped cannabis (14). Metals are reported among people who smoke cannabis without tobacco (15). "

Deviation from pre-registration in adjusting for cannabis use in follow-up models. If cannabis use attenuated the relationship with vaping or smoking, so observed differences could be mediated by cannabis. This is in discussion but could be mentioned earlier.

We have added extra detail on the association between cannabis, psychosis and toxicant exposure to the introduction.

"Cannabis use is also substantially greater among people with psychosis than without (15). Cannabis use, with and without tobacco, can also expose people to harmful toxicants. High levels of VOCs have been reported in the bio-samples of participants who smoke cannabis (16,17) and are also reported in the aerosol of vaped cannabis (18). Metals are reported among people who smoke cannabis with and without tobacco (19). There is little current evidence on the levels of tobacco toxicants that people with psychosis who smoke are exposed to and if these are affected by patterns of use or co-use of cannabis."

Any replication samples from UK or Europe, if nicotine/cannabis comorbidity is indeed lower?

Unfortunately, we are not aware of any biomarker studies similar to PATH in the UK or Europe to provide comparable evidence.

Is an implication that increased cadmium and uranium among EoP participants is mediated by cannabis, given the results comparing model 2 and model 3?

Thank you for highlighting this omission. We have added a comment on this to the discussion around cannabis. While cadmium was not associated with cannabis use, the association between uranium

and EoP was moderated by cannabis use. We have also added in the implications that cannabis harm reduction interventions are needed among this population to reduce toxicant exposure.

"Past 30-day cannabis use was associated with levels of lead, uranium, and most VOCs. These findings are consistent with previous research reporting high levels of VOCs in bio-samples of participants who smoke cannabis (16,17). Specifically, associations between EoP, uranium, and the VOC HPMM, lost significance when adjusting for cannabis use. This suggests that greater cannabis use among people with EoP contributes to higher exposure of these toxicants among this population."

"Overall, toxicant exposure was substantially lower among people with EoP who vaped compared to people with EoP who smoked. This supports the harm reduction potential of the use of e-cigarette as a smoking cessation tool for people with EoP, particularly as we found that they are exposed to significantly higher levels of cadmium and some VOCs due to their heaviness of tobacco smoking than people who smoke without EoP. Greater rates of cannabis consumption also contributed to higher levels of toxicant exposure. These findings emphasise the need for targeted stop-smoking support and cannabis harm reduction interventions, for this community to reduce toxicant exposure and subsequent disease. "
